# Mitochondrial antiviral signaling protein: a potential therapeutic target in renal disease

**DOI:** 10.3389/fimmu.2023.1266461

**Published:** 2023-10-12

**Authors:** Meng Wu, Zhiyin Pei, Guangfeng Long, Hongbing Chen, Zhanjun Jia, Weiwei Xia

**Affiliations:** ^1^ Department of Clinical Laboratory, Children’s Hospital of Nanjing Medical University, Nanjing, China; ^2^ Department of Nephrology, Children’s Hospital of Nanjing Medical University, Nanjing, China; ^3^ Jiangsu Key Laboratory of Pediatrics, Nanjing Medical University, Nanjing, China

**Keywords:** MAVS, renal disease, innate immune, NLRP3, inflammation

## Abstract

Mitochondrial antiviral signaling protein (MAVS) is a key innate immune adaptor on the outer mitochondrial membrane that acts as a switch in the immune signal transduction response to viral infections. Some studies have reported that MAVS mediates NF-κB and type I interferon signaling during viral infection and is also required for optimal NLRP3 inflammasome activity. Recent studies have reported that MAVS is involved in various cancers, systemic lupus erythematosus, kidney diseases, and cardiovascular diseases. Herein, we summarize the structure, activation, pathophysiological roles, and MAVS-based therapies for renal diseases. This review provides novel insights into MAVS’s role and therapeutic potential in the pathogenesis of renal diseases.

## 1 Introduction

The innate immune system plays the essential role in the initial defense mechanism against invading pathogens, serving as the frontline of protection. To mount an effective response, it relies on a sophisticated network of signaling pathways and molecules. Mitochondrial antiviral signaling protein (MAVS) is a key innate immune adaptor on the outer mitochondrial membrane that is required to defend against RNA viral stimulation (1). MAVS is also known as the IFN-β promoter stimulator (IPS-1) (2), caspase activation recruitment domain (CARD) adaptor inducing IFN-β (Cardif) (3), and virus-induced signaling adaptor (VISA) (4). Upon viral recognition, MAVS acts as a signaling hub, orchestrating a series of downstream events that activate the production of antiviral cytokines and interferons. Through its involvement in these intricate signaling cascades, MAVS contributes to the clearance of viral infections and maintenance of immune homeostasis (5). However, increasing evidence indicates that viruses can maintain survival and replication by disturbing multiple points in the MAVS signaling pathway to escape the host antiviral response during long-term coexistence. Furthermore, cells possess a strict regulatory mechanism to prevent spontaneous aggregation of MAVS and maintain immune homeostasis in the resting state (6). Owing to its dual role in antiviral signal transduction and immune homeostasis, the delicate regulation of MAVS has emerged as a central target for cell function and antiviral response. MAVS not only acts as a central hub in orchestrating the innate host response through the induction of antiviral and inflammatory pathways but is also involved in coordinating cellular damage and metabolic functions (7). Recent studies have shown that MAVS is involved in the occurrence and development of many diseases such as cancer (8, 9), systemic lupus erythematosus(SLE) (10), cardiovascular diseases(CVD) (11), acute kidney injury (AKI) (12), and chronic kidney disease(CKD) (13).

An estimated 1.2 million deaths of the global burden of disease can be attributed to kidney failure (14). Notably, patients and clinicians often underrecognize AKI because of limited diagnostic methods and rapid disease progression (15). Worldwide estimates indicate that CKD is the 16th leading cause of years of life lost, directly affecting global morbidity and mortality by increasing the risk of hypertension, diabetes, and CVD (16). The pathophysiological mechanisms underlying AKI and CKD are complex, involving many pathological processes, including glomerular injury, tubular injury, epithelial cell damage, pyroptosis, inflammation, vascular dysfunction, and tubulointerstitial fibrosis. Experimental studies have focused on the pivotal role of MAVS signaling in regulating kidney disease progression (16), indicating the potential of MAVS as a therapeutic approach for managing kidney disease. This review focuses on the role of MAVS in the etiology of renal dysfunction and the pathogenesis of kidney disease.

## 2 Molecular structure of MAVS

**Figure 1:**
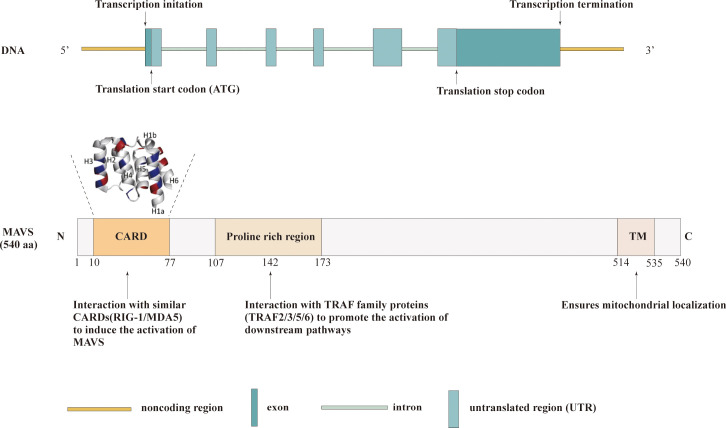
Structures of MAVS. Schematic representation illustrating the three functional domains of MAVS: the CARD domain, proline-rich region, and TM.

The MAVS gene is located on chromosome 20 in humans and consists of multiple exons and introns. The primary transcript undergoes alternative splicing, generating several isoforms with distinct functional properties. The full-length MAVS protein is a cytosolic protein comprising 540 amino acids. It contains several conserved domains, including an N-terminal caspase activation and recruitment domain(CARD), a central proline-rich region(PRR) and a C-terminal transmembrane domain(TM) (17). The N-terminal CARD domain of MAVS is responsible for protein-protein interactions, enabling MAVS to associate with other signaling molecules in the cytoplasm. This domain serves as a critical platform for the assembly of signaling complexes involved in antiviral signaling. Upon viral infection, the CARD domain of MAVS interacts with the CARD domains of retinoic acid-inducible gene I (RIG-I) or melanoma differentiation associated gene 5 (MDA5), initiating downstream signaling cascades (18). In addition, the MAVS-CARD domain is involved in caspase activation and has six helical structures, including H1–H6. Furthermore, helix1 is divided into two smaller helices, H1a and H1b. H1a, H3, and H4 form hydrophobic positive surfaces, whereas H2 and H6 form hydrophilic negative surfaces on the opposite side (19). The central proline-rich region of MAVS contains multiple conserved motifs, including a TRAF-interacting motif (TIM) and a proline-rich domain (PRD). These motifs enable MAVS to interact with various downstream effectors and adaptors, such as TNF receptor-associated factor (TRAF) proteins, facilitating the transmission of signals to downstream signaling pathways. Additionally, the proline-rich region acts as a scaffold for the recruitment of ubiquitin ligases, which play a crucial role in the activation and regulation of MAVS-mediated immune responses (20). The C-terminal transmembrane domain of MAVS anchors the protein to the outer mitochondrial membrane, allowing it to localize to the mitochondria-associated membranes (MAMs) (21). This localization is essential for MAVS to efficiently transmit signals from viral sensors to downstream effectors. The transmembrane domain also contributes to the formation of large MAVS aggregates, known as “signalosomes,” which serve as platforms for the amplification and propagation of antiviral signaling (20). The structure of the MAVS domain is shown in **Figure 1**.

## 3 The activation of MAVS

MAVS acts as a switch in immune signal transduction during viral infection. In the resting state, MAVS is kept silent. In the viral infection, RIG-I recognizes viral double-stranded RNA to activate RIG-I/MAVS antiviral functions, induce type I interferon production. As known many RNA viruses including dsRNA and ssRNA viruses produced double-stranded RNA (dsRNA) during replication to activate RIG-I/MAVS antiviral signaling. A list of some RNA viruses that have been reported to activate MAVS signaling was shown in **Table 1** (22–29). Furthermore, MAVS filament formation reveals structural and functional variability after viral infection (30). Notably, it has been reported that the combination of the MAVS-CARD and RIG-CARD domains results in the formation of MAVS aggregates on the surface of mitochondria, which can efficiently convert endogenous MAVS into functional aggregates. These aggregates activate cytosolic signaling cascades to induce antiviral molecules (31). MAVS TM domain has been shown to be critical to mediate the dimerization of MAVS in the activation of interferon regulatory factor 3/7 (IRF3/7) and NF-κB (32). In addition, the MAVS-PRR domain can recruit proteins to form MAVS aggregates and activate downstream effectors (33).

**Table 1:** List of RNA viruses activated MAVS signaling.

RNA viruses	dsRNA or ssRNA	References
Reovirus	dsRNA	(22)
Rotavirus	dsRNA	(23)
Influenza A Virus	ssRNA	(24)
Sendai virus	ssRNA	(25)
Vesicular stomatitis virus	ssRNA	(26)
Japanese Encephalitis Virus	ssRNA	(27)
Hepatitis C Virus	ssRNA	(28)
SARS-CoV-2	ssRNA	(29)

## 4 The antiviral signaling pathway of MAVS

Several studies have reported activation of the MAVS pathway by its interacting proteins and downstream signaling events. A recent study on the downstream signaling pathways of MAVS revealed that it mediates the activation of NF-κB and IRF3 by viruses (34). In the dormant state, NF-κB is sequestered in the cytoplasm by inhibitory κB (IκB) family members (35). Moreover, under the detection of viral RNA, MAVS then recruits IκB kinases IKKα, IKKβ, and IKKγ (an essential regulatory subunit, also called NF-kappa-B essential modulator (NEMO)) to induce the phosphorylation of IκB.

Phospho-IκB are recognized and ubiquitinated by ubiquitin ligases and subsequently degraded by the proteasome. Then, NF-κB translocates into the nucleus and binds to enhancers/promoters of target genes, activating the proinflammatory response (36). MAVS is a scaffold protein involved in activation upon RIG-I activation, which recognizes MAVS-interacting proteins in the RIG-I-mediated intracellular viral detection pathway. Lee et al. showed that mitochondrial MAVS recruits the signaling molecule TRAF6, activating NF-κB signaling pathways (37). Notably, K48-linked polyubiquitination of TRAF6 and NEMO is critical for NF-κB activation (38). Furthermore, Michallet et al. indicated that TRADD, a crucial adaptor of tumor necrosis factor receptor (TNFRI) and Fas-associated death domain (FADD), together with receptor-interacting protein (RIP) 1, play a crucial role in MAVS -induced NF-kB activation by the E3 ubiquitin ligase RIP1-bound TRAF2 (39). In addition, MAVS interacts with the Toll-interleukin-1 receptor domain-containing adaptor, inducing IFN-β (TRIF) in TLR-independent viral recognition pathways. The N-terminal- and C-terminal regions of TRIF are associated with TRAF6 and RIP1, respectively. RIP1 is further modified by K63 ligand ubiquitination to activate the downstream molecule TAK1. The formation of the TRAF6-RIP1-TAK1 complex plays a positive role in MAVS-induced NF-κB activation (40).

Viral components induce strong IRF3 activation through the Toll-like receptor (TLR) and RIG-I-like receptor (RLR) families. During the induction of IFNs, MAVS recruits TANK-binding kinase 1(TBK1) and inhibitor of kB kinase (IKKϵ), two primary kinases functionally that have been shown to phosphorylate the regulatory factor 3 and 7 (IRF3/IRF7), respectively. Furthermore, phosphorylated factors that translocate into the nucleus are critical for IFN production (41).

Upon RIG-I activation, MAVS activates the downstream pathways by recruiting related proteins. Stimulator of interferon genes (STING) (also known as MITA, MPYS, or ERIS) is an essential adaptor protein in the DNA recognition signaling pathway. The MAVS-STING interaction plays a vital role in mitochondria-mediated antiviral innate immune response (42). Upon viral RNA stimulation, the STING N-terminal fragment binds to the MAVS-CARD domain and interferon regulatory factor 3 (IRF3) to form the MAVS-STING and STING-IRF3 complexes, respectively. STING dimerization leads to the interaction of these two complexes and the activation of the IRF signaling pathway (43).

The Matrix Protein 2 (M2) is expressed by the influenza A virus upon infection, which plays a critical role in the viral replication and transmission cycle by serving as a multifunctional mediator of various processes (44). M2 protein has a proton-selective ion channel activity, which is the target of antiviral drug. M2 protein contains specific structural domains that enable it to interact with MAVS on mitochondria and positively regulating MAVS-mediated innate immunity. Importantly, M2 enhances MAVS signaling via increasing ROS production (45).

**Figure 2:**
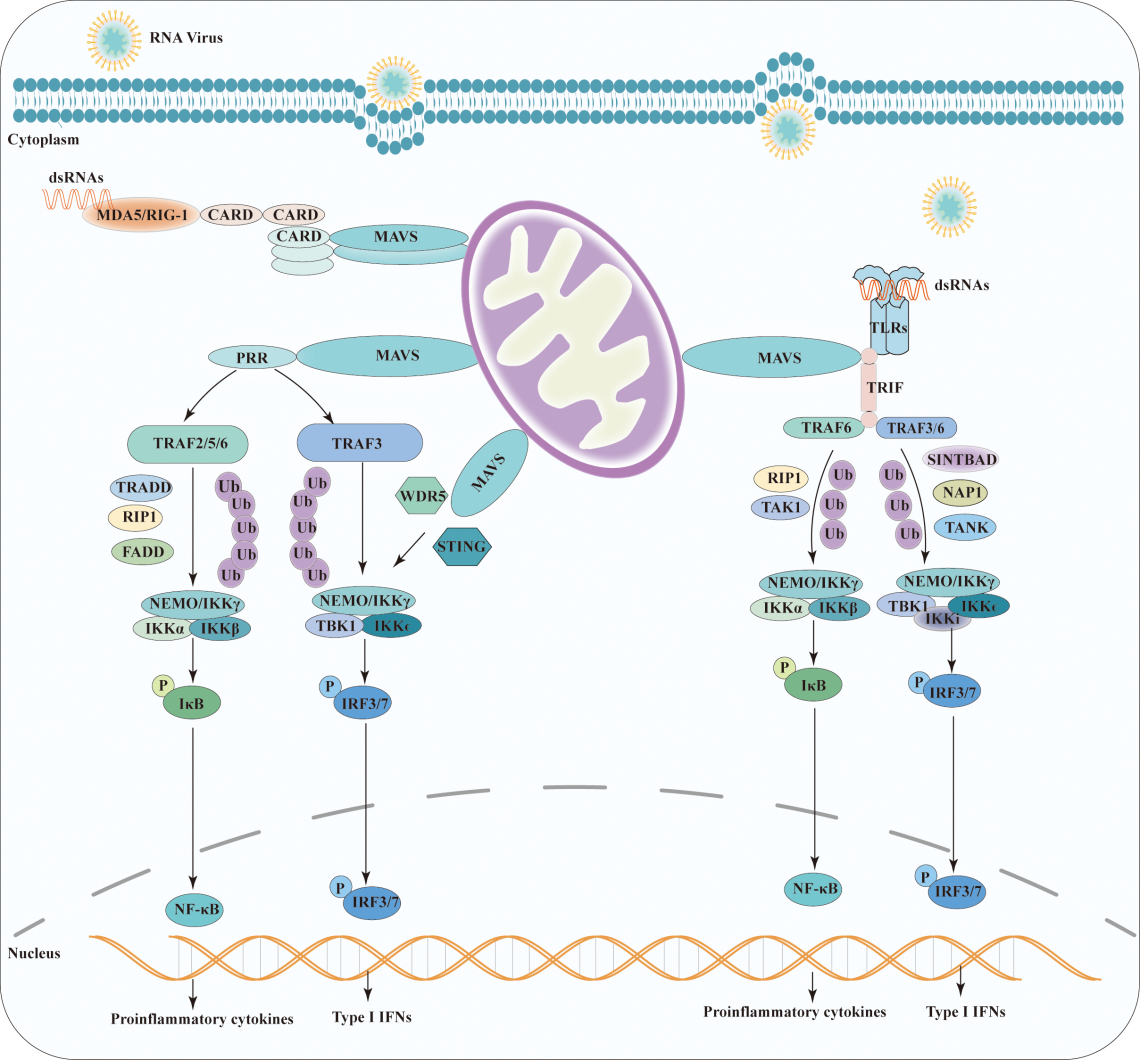
Schematic representation of the antiviral signaling pathway model of MAVS. Viruses induce MAVS activation through TLR and RLR families. Homophilic interactions between CARD domains enable RIG-I and MDA5 to interact with MAVS. Upon TLR stimulation, MAVS triggers downstream signaling cascades by recruiting TRAF and TRIF. MAVS activates two cytosolic protein kinase complexes, leading to the production of immune factors. The TBK1 complex phosphorylates IRF-3/7, promoting the transcription of IFN genes. Simultaneously, the IKK complex activates NF-kB, resulting in the production of proinflammatory cytokines.

WD repeat-containing protein 5 (WDR5), also known as SWD3 and BIG-3, is a member of the WD40 protein family. It has been identified as an important MAVS-associated component in the recruitment of RIG-I/STING to MAVS and the induction of type I IFNs. Further epistatic studies have shown that WDR5 functions upstream of TBK1 and downstream of RIG-I and MAVS (46). Notably, TRAF3 plays a critical role in the TLR-independent viral recognition pathways (47). Upon TLRs stimulation, MAVS activates downstream signaling of the TANK/TBK1/IKKi complex and NEMO the TANK/TBK1 complex by associating with the ubiquitin ligase TRAF3/TRAF6/TRAF5 during IFN induction (48, 49). Two adaptor proteins, SINTBAD and NAP1 and TANK, specifically bind to TBK1/IKKi during virus-induced IRF activation (50, 51). Alternatively, NEMO binds to polyubiquitinated TBK1, and the NEMO/TBK1 complex recruited to MAVS results in the activation of TBK1 and phosphorylation of IRF3 (52). In addition, k63-linked polyubiquitination of NEMO and TRAF3 is important for IRF3 activation in TLR-independent viral recognition pathways (38). All antiviral signaling pathways of MAVS are shown in **Figure 2**.

## 5 MAVS in kidney diseases

Accumulating evidence supports that MAVS is participated in the pathophysiology of human diseases. Here, we illustrate recent progress in comprehending the roles of MAVS in AKI, CKD, and other renal diseases, such as diabetic renal disease and hypertensive renal disease.

## 6 MAVS in AKI

AKI is a heterogeneous, broadly defined clinical syndrome characterized by high incidence and mortality (53). The most common causes of AKI include hypoxia, ischemia-reperfusion injury (IRI), sepsis, and nephrotoxic drugs (i.e., cisplatin and folic acid) (54). Many pathways are involved in AKI pathogenesis, including generating reactive oxygen species (ROS), inflammation, and activating several renal tubular epithelial cell death pathways (apoptosis, necrosis, ferroptosis, and pyroptosis) (55). MAVS, a key trigger in the activation of mitochondrial ROS (mtROS), is essential for optimal NLRP3 inflammasome activity and plays a vital regulatory role in host inflammation (56, 57). Previous studies demonstrated that NLRP3 is a key sensor of cell injury (58) and is expressed in proximal tubular epithelial cells in kidney disease (59). Furthermore, NLRP3 deficiency reduces renal tubular apoptosis in kidney injury and plays a role similar to that of ROS scavenger (59, 60). Moreover, NLRP3 or MAVS deletion attenuates mtROS production and mitochondrial membrane depolarization in renal tubular cells after renal hypoxia injury, indicating that MAVS is an indispensable and independent factor for inflammasome-independent NLRP3 activation (12). Another study indicated that NLRP3 deficiency afforded a significant renal protective effect against IRI in experimental mice, which could be linked with the inflammasome-independent role of NLRP3 in kidney injury (61). A later study revealed that MAVS was overexpressed in trichloroethylene (TCE)-sensitized positive mouse renal tubular cells and, mtROS inhibitor Mito TEMPO significantly decreased the expression of MAVS and the assembly of the NLRP3 inflammasome, suggesting that MAVS associates with NLRP3 and induces the activation of the NLRP3 inflammasome, finally contributing to tubular reabsorption dysfunction and kidney injury (62). Pinto et al. reported that West Nile virus (WNV) infection triggers signaling through MAVS, causing massive proinflammatory cytokine production that can result in sepsis and AKI in mice lacking type I IFN receptor gene (IFNAR) expression only in myeloid cell subsets (63).

Cisplatin, an anti-cancer drug, can cause AKI. However, the direct role of MAVS in cisplatin-induced AKI has not been elucidated. A previous study reported that ovarian cancer (OC) with high MAVS expression was remarkably less responsive to cisplatin and paclitaxel than those with low MAVS expression. Thus, MAVS appears to be a predictive biomarker of poor prognosis in OC (8). Additionally, several studies have indicated that the NLRP3 inflammasome represents a critical player in the pathogenesis of cisplatin-induced AKI (64–67). However, *Kim* et al. study reported that the kidney injury biomarkers, such as blood urea nitrogen, serum creatinine, tubular apoptosis score, and acute tubular necrosis score, were not statistically different between NLRP3 knockout (NLRP3^−/−^) mice and wild-type mice with cisplatin-induced AKI, which suggests that the NLRP3 inflammasome is not involved in cisplatin-induced AKI (68). The role of the NLRP3 inflammasome in cisplatin-induced AKI warrants further investigation. These findings suggest that modulating MAVS activity holds therapeutic potential for preventing or treating AKI.

## 7 MAVS in CKD

CKD is currently defined as a progressive decline in renal function associated with high morbidity and mortality, eventually leading to end-stage renal disease (ESRD) (69). The pathological process of CKD involves various factors, including proteinuria, unilateral ureteral obstruction (UUO), hyperuricemia, aldosterone, and subtotal nephrectomy (SNx) (13, 60, 70). Furthermore, apoptosis of renal tubular cells after AKI is an important initial step in the AKI-to-CKD progression (71). In that regard, a previous study found that MAVS induces cell death in a dose-dependent manner because of its cytotoxic effect; MAVS-induced apoptosis mediates the activation of the caspase-dependent pathway (72). Proteinuria, which is a key driving factor of tubulointerstitial fibrosis, is an independent risk factor for CKD. Moreover, as major targets, mitochondria play a pivotal role in the albumin-induced apoptosis of human proximal tubule cells (73). In the pathology of CKD induced by albumin, the knockdown of MAVS in human renal tubular epithelial cells weakened the colocalization between MAVS and NLRP3, thereby alleviating cell pyroptosis and epithelial-mesenchymal transition (13). Recently, the NLRP3 inflammasome and mitochondria were shown to participate in the development of various types of CKD. In a UUO-treated mouse model, NLRP3 deletion attenuated renal injury by preventing apoptosis and ameliorating tubulointerstitial fibrosis (12). In nephrocalcinosis-related CKD, NLRP3 antagonists decrease proinflammatory and fibrotic macrophage subsets and prevent renal fibrosis (74). Another study showed that NLRP3 knockout mice exhibited reduced renal tubular apoptosis and phenotypic alterations after aldosterone infusion, indicating that the NLRP3 inflammasome plays a vital role in aldosterone-induced renal injury (60). These studies suggest that MAVS and its downstream factor, NLRP3, regulate CKD.

## 8 MAVS in other kidney diseases

**Figure 3:**
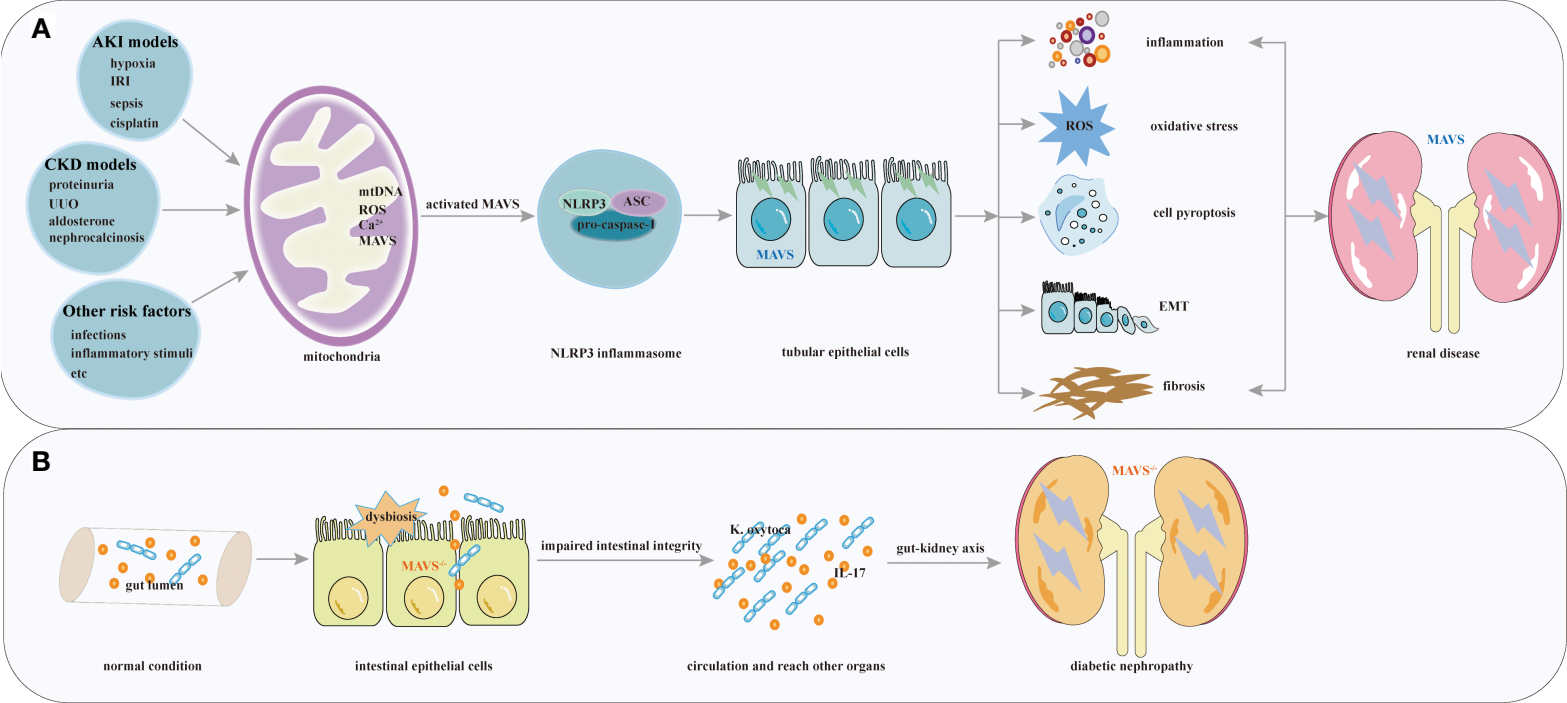
Overview of MAVS roles in renal diseases. **(A)** Multiple factors including hypoxia, IRI, sepsis, cisplatin, chronic kidney injury associated with proteinuria, UUO, hyperuricemia, aldosterone, and other risk factors activate MAVS and its downstream factor NLRP3 inflammasome. These activations stimulate processes such as inflammation, renal tubular epithelial cell injury, apoptosis, epithelial-mesenchymal transition, and tubulointerstitial fibrosis formation. **(B)** MAVS knockout contribute to the pathophysiology of diabetic nephropathy.

Mounting evidence indicates that NLRP3 has important effects on several kidney diseases, including pyelonephritis (75) and IgA nephropathy (76). These reports indicate that MAVS and its downstream factor, NLRP3, play diverse roles in renal disease by stimulating inflammation, renal tubular epithelial cell injury, apoptosis, epithelial-mesenchymal transition, and tubulointerstitial fibrosis. The function of MAVS signaling in kidney disease is shown in **Figure 3A**.

Diabetic kidney disease (DKD) is the leading cause of ESRD (77). A recent study showed that hyperglycemia might be associated with alterations in the epithelial barrier of the intestinal environment (78). The gut and the kidney are intricately linked through the gut-kidney axis. The gut microbiota and its metabolites affect kidney function and contribute to the development and progression of renal diseases. A previous study demonstrated that MAVS played a pivotal role in regulating and protecting intestinal permeability (79), and Linh et al. revealed elevated MAVS expression in diabetic kidneys. MAVS knockout diabetic mice had more severe glomerular and tubular injuries. In diabetic MAVS-knockout mice kidney, intestine-derived *Klebsiella oxytoca* and IL-17 production were observed because of impaired intestinal integrity. Furthermore, *Klebsiella oxytoca* supernatant or IL-17 promoted KIM-1 production in tubular epithelial cells, leading to renal injuries (80). These results suggest that systemic MAVS signaling confers a protective role in DKD. Which is different from the function of MAVS signaling in acute tubular necrosis and renal fibrosis (12, 56). The most straightforward explanation for these results would be that MAVS knockout contribute to the deleterious effects of hyperglycemia (81). Given the above, loss of kidney function can influence the intestinal barrier, and MAVS may play various roles within different microenvironment of the kidney. To date, interactions between intestinal and kidney specific deletion of MAVS have not been validated in kidney disease, and the role of MAVS in kidney tissue await further exploration. These results suggest that MAVS signaling, which is associated with regulating intestinal homeostasis, plays a protective role for DKD. The protective function of MAVS in kidney disease is shown in **Figure 3B**.

## 9 MicroRNA-mediated MAVS therapies

MicroRNAs, small non-coding RNA molecules, are pivotal in post-transcriptional gene expression regulation. They exert their influence by binding to mRNA molecules, subsequently guiding the RNA-induced silencing complex (RISC) to either degrade the target mRNA or impede its translation (82). Several miRNAs that potentially regulate the expression or function of MAVS have been identified. These miRNAs may modulate antiviral immune responses by targeting MAVS. The potential use of miRNAs and MAVS as therapeutic agents holds promise for the treatment of viral infections and other diseases.

MicroRNA-125a has been reported to target the MAVS 3’-UTR during viral infection, negatively regulating the antiviral response by inhibiting MAVS expression, leading to reduced production of type I interferons (83). Additionally, miR-125a and -b have recently been proven to bind directly to the endogenous 3’-UTR of MAVS, thereby downregulating the subsequent induction of type I and III IFNs. Furthermore, microRNA-125a identifies potential therapeutic options for the prevention and/or treatment of Influenza A viral infection in chronic obstructive pulmonary disease (84). Hou et al. reported that miR-3470b directly targets MAVS 3’-UTR in Bovine ephemeral fever virus infection. In that study, the authors discovered that the overexpression of miR-3470b resulted in a marked decrease in MAVS at both the transcriptional and protein levels, thereby suppressing the antiviral response (85). Further, miR-3570 negatively regulates rhabdovirus-triggered production of type I interferons by targeting MAVS in miiuy croaker macrophages, thus facilitating viral replication (86). Intriguingly, miR-22 directly targets and represses MAVS expression, which inhibits poly(I:C)-triggered production of type I interferons and inflammatory cytokines (87). Taken together, these findings suggested that specific miRNAs targeting MAVS may be a potential strategy for the management of renal diseases. All the miRNAs are listed in **Table 2**.

**Table 2:** List of several miRNAs that directly target MAVS.

MicroRNAs	Directly target region	References
microRNA-125a/b	MAVS 3’-UTR	(83, 84)
microRNA-3470b	MAVS 3’-UTR	(85)
microRNA-3570	MAVS 3’-UTR	(86)
microRNA-22	MAVS 3’-UTR	(87)

## 10 Inhibitory regulatory of MAVS signaling

MAVS acts as a key protein involved in kidney diseases. Therefore, understanding and inhibiting MAVS and its downstream signaling pathways have significant importance in management of renal diseases.

Recent studies have shown that several E3 ubiquitin ligases, including TRIM25, RNF5, MARCH5, Smurf1, Smurf2, NLRX1, RACK, OTUD1, OTUD4 and AIP4, have been identified to target MAVS for ubiquitination and subsequent degradation through Lys48-linked deubiquitination (88–96). Distinguishing from the above E3s, YOD1 suppress MAVS aggregation through Lys63-linked deubiquitination (97). At the meanwhile, MARCH8 acts as a negative regulator of the MAVS pathway by K27-linked deubiquitination. promoting MAVS degradation (98). The MAVS pathway is also regulated by viral proteases through cleavage of MAVS. For example, NS3/4A, CVB3 2Apro and PB1-F2 cleave MAVS, disrupting the downstream signaling cascade (99–101). RNF34, HFE, and Tetherin degrades MAVS complexes through autophagy (98, 102, 103). PSMA7 serves as a negative regulator of the MAVS for proteasome-dependent degradation (104). The inhibitory regulator of MAVS itself are listed in **Table 3**.

**Table 3:** List of several proteins that target MAVS protein itself.

Regulatory mechanism	Regulatory factor	Target in MAVS Signaling	References
K48-dependent ubiquitination	TRIM25, RNF5, MARCH5, Smurf1, Smurf2, NLRX1, RACK1, OTUD1, OTUD4, AIP4	MAVS protein	(88–96)
K63-dependent ubiquitination	YOD1	MAVS protein	(97)
K27-dependent ubiquitination	MARCH8	MAVS protein	(98)
Cleavage by viral proteases	NS3/4A, CVB3 2Apro, PB1-F2,	MAVS protein	(99–101)
Autophagy	RNF34, HFE, Tetherin	MAVS protein	(98, 102, 103)
Proteasome-dependent degradation	PSMA7	MAVS protein	(104)

Besides above proteins acting on MAVS itself, there are some small molecule inhibitors and natural products acts on MAVS downstream signaling. Several small molecule inhibitors, such as MRT67307, BX795 and BAY11-7082, targets MAVS downstream effector, TBK1, thereby inhibiting MAVS signaling (105–108). Besides, some natural compound (curcumin and resveratrol) derived from turmeric exhibits inhibitory effects on MAVS downstream signalling (109, 110). It has been shown to reduce MAVS-mediated inflammation and immune response in various viral infection models. The inhibitory regulator of MAVS downstream signaling are listed in **Table 4**.

**Table 4:** List of several inhibitors that target MAVS down streaming signaling.

Inhibitors	Target in MAVS down streaming signaling	References
Small Molecule Inhibitors		
MRT67307	inhibiting of TBK1	(105)
BX795	inhibiting of TBK1/IKK	(106)
BAY11-7082	inhibiting of NF-κB	(107, 108)
Natural Compounds		
Curcumin	Inhibiting of RIG-I pathway and IRF3 or NF-κB protein expression	(109)
Resveratrol	inhibiting of type I IFN production	(110)

These negative regulation helps to terminate MAVS signaling and prevent prolonged inflammation immune activation, which could be used for treatment of kidney diseases.

## 11 Conclusions and perspectives

In conclusion, as a critical adaptor protein, MAVS plays a central role in innate immunity and the pathological processes involved in renal disease. MAVS recruits its interacting proteins and downstream molecules upon viral infection to activate NF-κB and IRF signaling, which plays a crucial role in the mitochondria-mediated antiviral innate immune response. In addition, MAVS is required for optimal NLRP3 inflammasome activity, and the expression of MAVS and NLRP3 is mainly localized in tubular epithelial cells. Several studies have demonstrated the role of MAVS and NLRP3 inflammasomes in various experimental models of renal disease, including IRI, hypoxic injury, sepsis, cisplatin-induced AKI, UUO, albumin overload-induced tubular injury, nephrocalcinosis-related CKD, and DKD. These studies revealed that MAVS and its downstream factor, NLRP3, may provide a promising strategy for treating renal disease.

To date, only phosphorylation and polyubiquitination were verified to regulate the activation of MAVS-mediated signaling, whether MAVS could be modified by other manners such as glycosylation, and the effects of these manners on MAVS activation still require further exploration. However, the existing literature on the regulation of MAVS signaling mainly focuses on animal or cell experimental models of renal disease. Relatively few studies have reported MAVS expression levels in clinical patients. Moreover, direct evidence of the role of MAVS in the pathogenesis of renal disease is lacking. The underlying mechanisms of MAVS in different renal diseases warrant further in-depth investigation.

The current emphasis in MAVS expression research revolves around its potential as a clinical prognostic biomarker and therapeutic target, primarily within the field of cancer. The various applications and development of MAVS agonists and antagonists may provide new therapeutic strategies for future research in renal diseases. It is important to note that miRNA regulation can be complex and that the interactions between miRNAs and their target genes can vary depending on the cellular context and specific conditions. Therefore, further research is required to fully understand the regulatory networks involving miRNAs and MAVS in different biological settings.

## Author contributions

MW: Funding acquisition, Writing – original draft, Writing – review & editing. ZP: Writing – original draft, Writing – review & editing. GL: Writing – original draft, Writing – review & editing. HC: Conceptualization, Supervision, Writing – review & editing. ZJ: Conceptualization, Supervision, Writing – review & editing. WX: Conceptualization, Funding acquisition, Supervision, Writing – original draft, Writing – review & editing.
